# Adenovirus Type 7 Genomic-Type Variant, New York City, 1999

**DOI:** 10.3201/eid1001.020605

**Published:** 2004-01

**Authors:** Jennifer Ann Marie Calder, Dean D. Erdman, Joel Ackelsberg, Stephen William Cato, Vicki-Jo Deutsch, Anthony John Lechich, Barbara Susan Schofield

**Affiliations:** * Terence Cardinal Cooke Health Care Center, New York, New York, USA; † Centers for Disease Control and Prevention, Atlanta, Georgia, USA; ‡ New York State Department of Health, Albany, New York, USA

**Keywords:** acute respiratory disease viruses, adenovirus infections, adenoviruses human, adenoviridae, APC virus, disease outbreaks, outbreaks, communicable diseases, epidemics, infectious diseases

## Abstract

An outbreak of respiratory illness occurred in a long-term care facility in New York City. Investigation of the outbreak identified confirmed or suspected adenoviral infection in 84% of the residents from October 19 to December 18, 1999. Further identification by type-specific neutralization and restriction analysis identified a new genomic variant of adenovirus type 7.

Human adenoviruses are known to cause a variety of illnesses, including cystitis, diarrhea, intussusception, meningoencephalitis, epidemic keratoconjunctivitis, and encephalitis (*1*). Communitywide outbreaks of respiratory illness attributable to adenovirus, particularly serotypes 3, 4, 7, and 21, have also been described in civilian (*2*,*3*) and military (*4*) populations. Institutional outbreaks involving pediatric populations or persons with underlying pulmonary disease can be particularly severe, with high illness and death rates (*5*,*6*).

On November 27, 1999, three residents in the specialty hospital of a large long-term care facility in New York City were hospitalized with respiratory failure. When additional illnesses were identified, control measures and an outbreak investigation were initiated. We present the results of that investigation and document a novel genome type of adenovirus serotype 7 (Ad7) as the etiologic agent of the outbreak.

## The Study

 Chart reviews were conducted for all residents of the specialty hospital and retrospectively for persons who had died or were discharged in the previous 2 months. A confirmed case-patient was defined as a resident of the specialty hospital in whom new respiratory symptoms developed from October 19, 1999, to January 10, 2000, and who had a positive laboratory test for adenovirus or a histopathologic finding that was indicative of adenovirus infection. A suspected case-patient was defined as a resident of the specialty hospital in whom new respiratory symptoms developed from October 19, 1999, to January 10, 2000, and who tested negative for adenovirus or was not tested. A noncase-patient was defined as anyone without respiratory symptoms, regardless of test results, or anyone with respiratory symptoms and with an organism other than adenovirus identified through laboratory testing in the same period.

 Specimens initially were requested only from symptomatic residents. As the outbreak continued, we attempted to obtain specimens from all residents in an effort to identify carriers. Nasal washings or tracheal secretions were tested for respiratory syncytial virus (RSV), influenza A and B, parainfluenza 1, 2, and 3, and adenovirus antigens by a commercial immunofluorescence assay (VRK Bartels Viral Respiratory Screening and Identification Kit, Bartels Inc., Issaquah, WA) and by enzyme immunoassay (Directigen RSV, Becton Dickinson, Le Pont de Claix, France) for RSV. Specimens were also added to MRC5, RMK, and A549 cells, and the cells were monitored for cytopathic effect. Selected specimens were tested for adenovirus by polymerase chain reaction (PCR) assay, using adenovirus group- and Ad7-specific primer sets (*7*,*8*). Autopsied lung tissue specimens also were sent to the Centers for Disease Control and Prevention (CDC) for routine histopathologic examination.

Adenovirus isolates were typed by use of a microneutralization assay (*9*). Genome typing by restriction analysis was performed by using 12 restriction enzymes, *Bam*HI, *BcI*I, *Bg*I, *Bgl*II, *BstE*II, *Eco*RI, *Hin*dIII, *Hpa*I, *Sal*I, *Sma*I, *Xba*I , and *Xho*I, as previously described (*5*). Restriction patterns were interpreted by using the genome type classification scheme of Li and Wadell (*10*) and Li et al. (*11*). An additional 27 unrelated Ad7 community isolates collected between 1995 and 2000 were provided by several healthcare centers in the New York City area for comparison with the outbreak strains. Data were analyzed by using EpiInfo version 6.04b (CDC) for descriptive statistics.

Of the 50 residents in the specialty hospital from October 19, 1999, to January 10, 2000, 23 (46.0%) were females and 27 (54.0%) were males. Their ages ranged from 1 to 46 years (mean 11; median 10). The residents were located in two units: A with 21 (42.0%) and B with 29 (58.0%) residents. On average, the residents in B were younger than those in A (mean age 7 years [median 5; range 1-32] versus mean age 16 years [median 14; range 3-46]).

Of the 50 residents, 30 (60.0%) had confirmed cases of adenovirus infection, 12 (24.0%) had suspected cases, and 8 (16.0%) did not have adenovirus. Of the 30 patients with confirmed cases, 19 (63.3%) were hospitalized, and 5 died (confirmed case-fatality rate, 16.7%). Of the 12 patients with suspected cases, 7 (58.3%) were hospitalized and 2 died (suspected case-fatality rate, 16.7%). Of the eight noncase-patients, one person (12.5%) exhibited respiratory symptoms and was confirmed to be infected with herpes simplex virus; the other seven (87.5%) exhibited no respiratory symptoms, and three of six tested positive for adenovirus.

Confirmed case-patients were on average younger than suspected and noncase-patients (mean age 9 years [median 6; range 1-32] versus 12 years [median 12; range 1-25] and 13 years [median 12; range 1-6], respectively. More than 80% of confirmed and suspected case-patients had a tracheostomy, versus 37% of noncase-patients.

**Figure 1:**
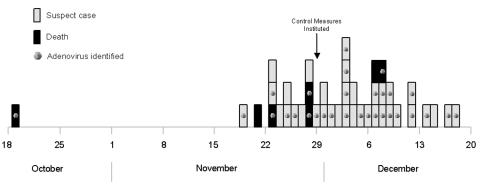
Epidemic curve showing onset date of illness for confirmed and suspected cases of adenovirus infection, New York City, 1999.

Symptom onset dates for residents with respiratory illness ranged from October 19, 1999, to December 18, 1999 (Figure 1). The Table describes the clinical characteristics of confirmed and suspected case-patients. The mean duration of illness for confirmed patients was 21 days (median 20; range 2-62) versus 28 days (median 22; range 6-93) for suspected case-patients.

**Table:** **Table.** Clinical characteristics of the confirmed and suspected adenovirus case-patients in a specialty hospital unit of a long-term care facility, New York City, October 19, 1999, to January 10, 2000^a^

Clinical characteristic	Confirmed (N = 30)	Suspected (N = 12)
Female	13 (43.3)	5 (41.7)
Male	17 (56.7)	7 (58.3)
Mean age, in y	9	12
Median (range)	6 (1–32)	12 (1–25)
Fever >38.3°C	30 (100.0)	11 (91.7)
Increased tracheal secretions	24 (80.0)	10 (83.3)
Chest congestion	23 (76.7)	11 (91.7)
Tracheitis	23 (76.7)	9 (75.0)
Pneumonia	18 (60.0)	7 (58.3%)
Wheezing	16 (53.3)	5 (41.2)
Cough	9 (30)	4 (33.3)
Nasal congestion	2 (6.7)	0
Runny nose	1 (3.3)	1 (8.3)

^a^Except for age, data are number of patients with the characteristic (%).

**Figure 2:**
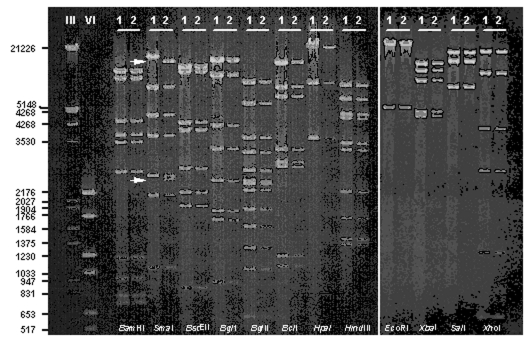
DNA fragment patterns obtained with selected restriction enzymes of representative outbreak (1) and community (2) Ad7 isolates resolved by gel electrophoresis with ethidium bromide staining. DNA markers III (λ*Hin*dIII/*Eco*RI) and VI (pBR328 *Bgl*I/*Hinf*I) were run simultaneously to facilitate fragment size estimates. Arrows highlight loss of 2,500- and 12,700-bp fragments and corresponding appearance of a new 15,200-bp fragment for the outbreak strain (1) as compared with the expected pattern for Ad7b (2).

Specimens from 48 (96.0%) of the 50 residents were submitted for testing. Of them, 26 (60.5%) of 43 were adenovirus positive by culture, 13 (54.2%) of 24 were positive by adenovirus group–specific PCR, 6 (22.2%) of 27 were positive by antigen testing, and 3 (100%) of 3 were positive by histopathology. Overall, 33 (68.8%) persons tested positive for adenovirus. Of 15 outbreak isolates submitted to CDC for further characterization, all were identified as serotype 7 by type-specific neutralization. Restriction analysis with 12 previously described restriction enzymes further identified all isolates as *Sma*I variants of genome type 7b (Figure 2). Restriction patterns obtained with 11 other enzymes were identical with those predicted for genome type 7b (*12*,*13*). Of the 28 additional Ad7 field isolates from the New York City area that were epidemiologically unrelated to the outbreak, 24 were identified as Ad7b and 4 as Ad7d2; none possessed the unique *Sma*I restriction pattern of the outbreak strain (data not shown).

## Conclusions

This outbreak illustrates the difficulty inherent in controlling the spread of adenovirus in a closed community and the potential for severe disease and death in persons with underlying respiratory disease. When both confirmed and suspected cases are included, a remarkably high attack rate of 84% was documented among the 50 residents of the specialty hospital, with 26 hospitalizations and 7 deaths. The epidemiologic association and similar hospitalization and fatality rates for confirmed and suspected cases support our conclusion that most suspected cases likely had adenovirus infections.

 A possible explanation for the 1-month lapse between the initial case in October and the other cases may be that the virus was first introduced into the facility in October but was transmitted more efficiently during the colder months, when there were more indoor activities and crowding on the units. A previously published report of an outbreak in a chronic-care facility (*5*) identified younger age and tracheostomy as risk factors for adenovirus infection. The high attack rate in this outbreak prevented calculation of risk factors. However, we observed a higher percentage of confirmed cases among the younger residents and persons with a tracheostomy. Therefore, we conclude that the underlying chronic respiratory disease in this population facilitated the transmission of this pathogen and that Ad7b variant may represent an emerging risk to similar persons.

The Ad7b genome type is widely distributed geographically and has been the predominant strain circulating in the United States since the late 1960s (*12*,*13*) (D. Erdman, pers. observation). Although genetic variants of Ad7b have been described using other restriction enzymes (*12*), to our knowledge, this is the first description of the *Sma*I variant of Ad7b. The novel *Sma*I pattern was most likely derived from the loss of the single restriction site that forms fragments 12,700 bp and 2,500 bp; loss of these two fragments corresponded with the appearance of a new 15,200-bp fragment. Failure to identify this variant in past studies or among recent Ad7s isolates circulating in the New York community suggests that it arose recently and supports the hypothesis that this outbreak resulted from nosocomial spread of the virus within the chronic care unit. Whether the genetic mutation(s) in the outbreak strain contributed to the severity of illness documented in these patients could not be determined.

Adenoviruses cause severe and often fatal respiratory disease in immunocompromised patients (*1*). A large outbreak of acute respiratory disease and pneumonia at a chronic mental health facility recently was attributed to adenovirus 35, a pathogen that has typically affected immunocompromised patients (*14*). Investigators speculated that immunity to Ad35 may have waned in the long-term residents, who may have been less likely to have been exposed to adenoviruses circulating in the community.

Recent outbreak investigations and surveillance studies (*15*) suggest that certain U.S. populations at risk may be experiencing an increase in acute respiratory disease caused by adenoviruses. In settings such as long-term care facilities that house patients with susceptible underlying conditions, infection with virulent adenoviral strains should be considered when patients are seen with sudden respiratory disease, and appropriate control measures should be implemented quickly, pending pathogen identification.

### Acknowledgments

 We thank the institutions that kindly provided adenovirus isolates for this study and the persons who performed diagnostic testing: Angela Rendo, Syed Sherazi, Gary Leonardi, Iqbal Poshni, Nando Chatterjee, Sherif Zaki, and Wun-Ju Sheif.
